# MSTN, mTOR and FoxO4 Are Involved in the Enhancement of Breast Muscle Growth by Methionine in Broilers with Lower Hatching Weight

**DOI:** 10.1371/journal.pone.0114236

**Published:** 2014-12-01

**Authors:** Chao Wen, Yueping Chen, Ping Wu, Tian Wang, Yanmin Zhou

**Affiliations:** College of Animal Science and Technology, Nanjing Agricultural University, Nanjing, Jiangsu, China; Kobe University, Japan

## Abstract

Broilers with lower hatching weight (HW) present poorer performance than those with high HW, but there is limited research on the growth regulation of broilers with lower HW. The objective of this study was to investigate the effect of dietary methionine (Met) levels on the growth performance and breast muscle yield of broilers with different HW and underlying mechanisms. A total of 192 one-day-old Arbor Acres broiler chicks with different HW (heavy: 48.3±0.1 g, and light: 41.7±0.1 g) were allocated to a 2×2 factorial arrangement with 6 replicates of 8 chicks per replicate cage. Control starter (1–21 d) and finisher (22–42 d) diets were formulated to contain 0.50% and 0.43% Met, respectively. Corresponding values for a high Met treatment were 0.60% and 0.53%. Light chicks had lower body weight gain (BWG) and breast muscle yield than heavy chicks when the broilers were fed the control diets. High Met diets improved BWG, gain to feed ratio and breast muscle yield in light but not heavy chicks. Decreased DNA content and increased RNA/DNA and protein/DNA ratios in breast muscle were induced by high Met diets only in light chicks. MSTN mRNA level was decreased by high Met diets only in light chicks, and this decrease was accompanied by a significant increase in MSTN gene exon 1 methylation. In addition, high Met diets increased mTOR phosphorylation, but decreased FoxO4 phosphorylation in breast muscle of light chicks. In conclusion, the BWG and breast muscle yield of light chicks were improved by increasing dietary Met levels probably through alterations of MSTN transcription and phosphorylation of mTOR and FoxO4.

## Introduction

One of the most important factors affecting broiler performance is hatching weight (HW) of chicks [1], which is about 68% of egg weight [2]. Egg size increases with breeder age, thus chicks from older breeders have higher HW than those from young breeders. Several recent studies have shown that broilers with higher HW present better performance [3], [4]. Sklan et al. [5] found that chicks with higher HW improved marketing weight by enhancing breast muscle growth. However, there is limited research on the growth regulation of broilers with lower HW.

Skeletal muscle growth is regulated by myostatin (MSTN) and related signaling pathways, such as extracellular signal-regulated kinase (ERK), mechanistic target of rapamycin (mTOR) and forkhead box O (FoxO) [6], [7], [8]. The mRNA expression and DNA methylation of MSTN have been reported to be affected by dietary Met [9], which is the first limiting amino acid in broiler diets. Several reviews have suggested that Met may act as regulators of protein metabolism and associated signaling pathways [10], [11]. The positive effect of Met on breast muscle yield of broilers has been demonstrated [12], thus it can be hypothesized that increasing dietary Met level may improve performance and breast muscle growth of broilers with lower HW by regulating MSTN expression and related signaling pathway. Recently, we reported that increased mTOR and decreased FoxO4 mRNA levels were induced by high Met diets in broiler chicks with lower HW [13], but whether the phosphorylation of these proteins is affected by dietary Met remains unclear. Therefore, the objective of this study was to investigate the effect of dietary Met levels on growth performance, breast muscle yield, mRNA expression and DNA methylation of MSTN and related signaling pathways in broilers with different HW.

## Materials and Methods

### Experimental design

All procedures were approved by Nanjing Agricultural University Institutional Animal Care and Use Committee. A total of 192 one-day-old Arbor Acres broiler chicks with different HW (heavy: 48.3±0.1 g, and light: 41.7±0.1 g) were allocated to a 2×2 factorial arrangement with 6 replicates of 8 chicks (4 males and 4 females) per replicate cage. Control starter (1–21 d) and finisher (22–42 d) diets were formulated to contain 0.50% and 0.43% Met, respectively, according to NRC (1994) requirements for broilers (Table 1). A high Met treatment (0.60 and 0.53% Met during the starter and finisher phase, respectively) was formulated by increasing the level of DL-Met (99%; Adisseo Inc., Antony, France) in the diets. Chicks were allowed free access to mash feed and water in 3-layer cages in a temperature-controlled room with continuous lighting. The temperature of the room was maintained at 32 to 34°C for the first 3 d and then reduced by 2 to 3°C per week to a final temperature of 20°C. At 42 d of age, the broilers were weighed after feed deprivation for 12 h and feed intake (FI) was recorded by replicate to calculate body weight gain (BWG) and gain to feed ratio (G:F). Mortality was also recorded.

**Table 1:** Composition and nutrient level of diets (as fed basis).

	1–21 d		22–42 d	
Items	CM	HM	CM	HM
Ingredient (%)				
Corn	57.0	57.0	61.9	61.9
Soybean meal	31.3	31.3	25.6	25.6
Corn gluten meal	3.9	3.9	4.3	4.3
Soybean oil	3.1	3.1	3.8	3.8
Dicalcium phosphate	1.8	1.8	1.6	1.6
Limestone	1.3	1.3	1.2	1.2
L-Lysine, HCl	0.15	0.15	0.2	0.2
DL-Methionine	0.15	0.25	0.1	0.2
Sodium chloride	0.3	0.3	0.3	0.3
Vitamin and mineral mix^a^	1.0	1.0	1.0	1.0
Calculated nutrient content				
Metabolizable energy (MJ/kg)	12.69	12.71	13.10	13.12
CP (%)	21.52	21.58	19.71	19.77
Lysine (%)	1.14	1.14	1.04	1.04
Methionine (%)	0.50	0.60	0.43	0.53
Total sulfur amino acids (%)	0.85	0.95	0.76	0.86
Calcium (%)	1.00	1.00	0.90	0.90
Non-phytate phosphorus (%)	0.46	0.46	0.42	0.42
Analyzed nutrient content				
DM (%)	90.40	90.52	89.08	88.73
CP (%)	21.76	21.69	19.23	19.54
Ether extract (%)	5.96	5.89	5.67	5.75
Lysine (%)	1.18	1.15	1.02	1.06
Methionine (%)	0.51	0.63	0.40	0.55
Total sulfur amino acids (%)	0.89	0.97	0.73	0.82
Methionine/total sulfur amino acids	0.57	0.65	0.55	0.67
Methionine/lysine	0.43	0.55	0.39	0.52

a Providing the following per mg/kg diet: retinyl acetate, 3.44; cholecalciferol, 0.075; all-rac-α-tocopherol acetate, 30; menadione, 1.3; thiamin, 2.2; riboflavin, 8; nicotinamide, 40; choline chloride, 600; calcium pantothenate, 10; pyridoxine·HCl, 4; biotin, 0.04; folic acid, 1; cobalamin, 0.013; Fe (as FeSO_4_.H_2_O), 80; Cu (as CuSO_4_.5H_2_O), 8; Mn (as MnSO_4_.H_2_O), 110; Zn (as ZnO), 65; I (as KIO_3_), 1.1; Se (as Na_2_SeO_3_), 0.3.

### Sample collection

At 42 d of age, 1 male broiler from each replicate was randomly selected and weighed after feed deprivation for 12 h. Broilers were killed by cervical dislocation, and the whole breast (including pectoralis major and minor) muscle was weighed. Then samples were collected from pectoralis major muscle and stored in liquid nitrogen until analysis.

### Measurement of protein and nucleic acids in breast muscle

The contents of DNA and RNA in muscle samples were measured according to the procedures of Johnson and Chandler [14]. Tissue protein content was determined by the colorimetric method of Bradford [15].

### Real-time PCR

Total RNA was isolated from muscle samples as previously described by Wen et al. [16], using RNAiso Reagent (Takara Bio, Inc., Dalian, Liaoning, China). Reverse transcription of total RNA was completed using a reagent kit (PrimeScript RT Reagent Kit; Takara Bio, Inc.). The geometric means of glyceraldehyde 3-phosphate dehydrogenase (GAPDH) and β-actin were used to normalize MSTN mRNA expression as recommended by Vandesompele et al. [17]. The primers for MSTN, β-actin and GAPDH were synthesized according to Chen et al. [18], Kogut et al. [19], and Wang et al. [20], respectively. Quantification of mRNA was performed on a PCR system (ABI 7300 Real-Time PCR System; Applied Biosystems, Foster City, CA) using a kit (SYBR Premix Ex Taq II Kit; Takara Bio, Inc.), according to the instruction of the manufacturer. All reactions were run in triplicate, and the average values were obtained to calculate the relative mRNA level of MSTN as previously described [21]. The MSTN mRNA level in heavy chicks fed the control diets was assigned a value of 1.

### DNA methylation assay

Total DNA was extracted from muscle samples using a kit (Genomic DNA Extraction Kit; Sangon, Shanghai, China) and modified using a kit (Methylcode Bisulfite Conversion Kit; Invitrogen, Carlsbad, CA). Bisulfite sequencing primers to amplify the exon 1 region of MSTN gene were synthesized as described by Liu et al. [9]. The Hotstart PCR (Agilent, Foster City, CA) was performed using the following cycling program: 95°C for 2 min, followed by 30 cycles of 95°C for 30 s, 56°C for 30 s, 72°C for 1 min, and a final step at 72°C for 7 min. Then PCR products were purified using a kit (QIAquick PCR Purification Kit; Qiagen, Hilden, Germany) and subcloned using a kit (TOPO TA Cloning Kit; Invitrogen). Finally the products were sequenced for comparison with the University of California Santa Cruz (Santa Cruz, CA) genome reference sequence to assess the methylation status of MSTN gene exon 1 region as previously described [22].

### Western blot

The muscle lysates were prepared as described by Wang et al. [23], and subjected to SDS-PAGE gel electrophoresis and western blot analysis using appropriate antibodies: mTOR, FoxO4, p-mTOR (Ser2448), p-FoxO4 (Ser193) and p-ERK1/2 (Thr202/Tyr204) were purchased from Cell Signaling Technology, Inc. (Danvers, MA), and ERK1/2 from Bioworld Technology, Inc. (St. Louis Park, MN). After washing, membranes were incubated with a secondary antibody (Rockland Immunochemicals, Gilbertsville, PA). The bands were visualized by infrared fluorescence using the Odyssey Imaging System (LI-COR) and quantified by Odyssey infrared imaging system software.

### Statistical analysis

Two-way ANOVA was employed to determine the main effects of HW and Met and their interaction using the general linear model procedure of SPSS software (version 16.0; SPSS Inc., Chicago, IL, USA). Differences among treatments were examined by one-way ANOVA using Duncan's multiple range test, which were considered significant at *P*<0.05. Data are presented as means and pooled standard error of the mean (SEM).

## Results

### Growth performance

Mortality was low (3%) and not related to treatment. Light chicks had lower BWG and FI than heavy chicks during 1–21 d, and the same pattern was observed in BWG during 22–42 and 1–42 d when the broilers were fed the control diets (Table 2). High Met diets improved BWG and G:F in light but not heavy chicks during 22–42 and 1–42 d, whereas FI had no significant difference.

**Table 2:** Broiler performance.

	Heavy		Light			P		
Item	CM	HM	CM	HM	SEM	HW	Met	HW × Met
1–21 d								
BWG (kg)	0.69^a^	0.68^a^	0.64^b^	0.67^ab^	0.01	0.008	0.578	0.048
FI (kg)	1.03	1.01	0.97	0.97	0.01	0.027	0.584	0.702
G:F	0.68	0.67	0.66	0.69	0.01	0.801	0.361	0.238
22–42 d								
BWG (kg)	1.63^a^	1.55^ab^	1.43^b^	1.66^a^	0.03	0.488	0.260	0.029
FI (kg)	3.25	3.04	3.02	3.14	0.06	0.548	0.717	0.152
G:F	0.50^ab^	0.51^ab^	0.48^b^	0.53^a^	0.01	0.725	0.015	0.087
1–42 d								
BWG (kg)	2.32^a^	2.23^ab^	2.07^b^	2.32^a^	0.03	0.265	0.237	0.016
FI (kg)	4.27	4.05	3.99	4.10	0.05	0.294	0.635	0.126
G:F	0.54^ab^	0.55^a^	0.52^b^	0.57^a^	0.004	0.721	0.007	0.049

Heavy −48.3±0.1 g; Light −41.7±0.1 g; CM - control methionine levels (0.50% and 0.43% methionine during 1–21 d and 22–42 d, respectively); HM - high methionine levels (0.60% and 0.53% methionine during 1–21 d and 22–42 d, respectively); HW - hatching weight.

BWG -body weight gain; FI - feed intake; G:F – gain to feed ratio.

SEM - standard error of the mean.

Within the same row, different superscripts indicate significant differences (*P*<0.05).

### Breast muscle yield and its DNA, RNA and protein contents

The breast muscle yield was greater in heavy chicks than in light chicks when both were fed the control diets, and lower DNA content and higher RNA/DNA were observed in breast muscle of heavy chicks (Table 3). High Met diets increased the yield of breast muscle and its RNA/DNA and protein/DNA ratios in light but not heavy chicks at 42 d of age. The DNA content in breast muscle was decreased by high Met diets only in light chicks, whereas RNA, protein content or RNA/protein did not differ among treatments.

**Table 3:** Breast muscle yield and its DNA, RNA and protein contents.

	Heavy		Light			P		
Item	CM	HM	CM	HM	SEM	HW	Met	HW × Met
Yield (%)	18.6^a^	18.0^ab^	17.2^b^	18.7^a^	0.2	0.487	0.318	0.042
DNA(mg/g)	1.75^b^	1.78^ab^	2.05^a^	1.59^b^	0.05	0.542	0.034	0.018
RNA(mg/g)	2.43	2.90	2.14	2.39	0.10	0.062	0.088	0.606
Protein(mg/g)	82.9	82.5	81.4	81.5	1.5	0.690	0.957	0.922
RNA/protein	0.030	0.035	0.027	0.030	0.001	0.125	0.131	0.659
RNA/DNA	1.39^a^	1.64^a^	1.05^b^	1.50^a^	0.05	0.032	0.003	0.348
Protein/DNA	47.7^ab^	46.7^ab^	40.7^b^	51.7^a^	1.4	0.709	0.079	0.039

Heavy −48.3±0.1 g; Light −41.7±0.1 g; CM – control methionine levels (0.50% and 0.43% methionine during 1–21 d and 22–42 d, respectively); HM - high methionine levels (0.60% and 0.53% methionine during 1–21 d and 22–42 d, respectively); HW - hatching weight.

SEM - standard error of the mean.

Within the same row, different superscripts indicate significant differences (*P*<0.05).

### Myostatin (MSTN) mRNA level and DNA methylation

High Met diets decreased mRNA level and increased methylation of gene exon 1 region of MTSN in light but not heavy chicks at 42 d of age, whereas no difference was observed when the broilers were fed the control diets (Table 4).

**Table 4:** Myostatin (MSTN) mRNA level and DNA methylation of gene exon 1 region.

	Heavy		Light			P		
Item	CM	HM	CM	HM	SEM	HW	Met	HW × Met
mRNA level	1.00^ab^	0.60^b^	1.45^a^	0.80^b^	0.09	0.088	0.011	0.506
Methylation (%)	33.3^ab^	29.6^ab^	20.7^b^	43.5^a^	2.5	0.900	0.079	0.020

Heavy −48.3±0.1 g; Light −41.7±0.1 g; CM – control methionine levels (0.50% and 0.43% methionine during 1–21 d and 22–42 d, respectively); HM - high methionine levels (0.60% and 0.53% methionine during 1–21 d and 22–42 d, respectively); HW - hatching weight.

SEM - standard error of the mean.

Within the same row, different superscripts indicate significant differences (*P*<0.05).

### Phosphorylation of ERK1/2, mTOR and FoxO4

**Figure 1:**
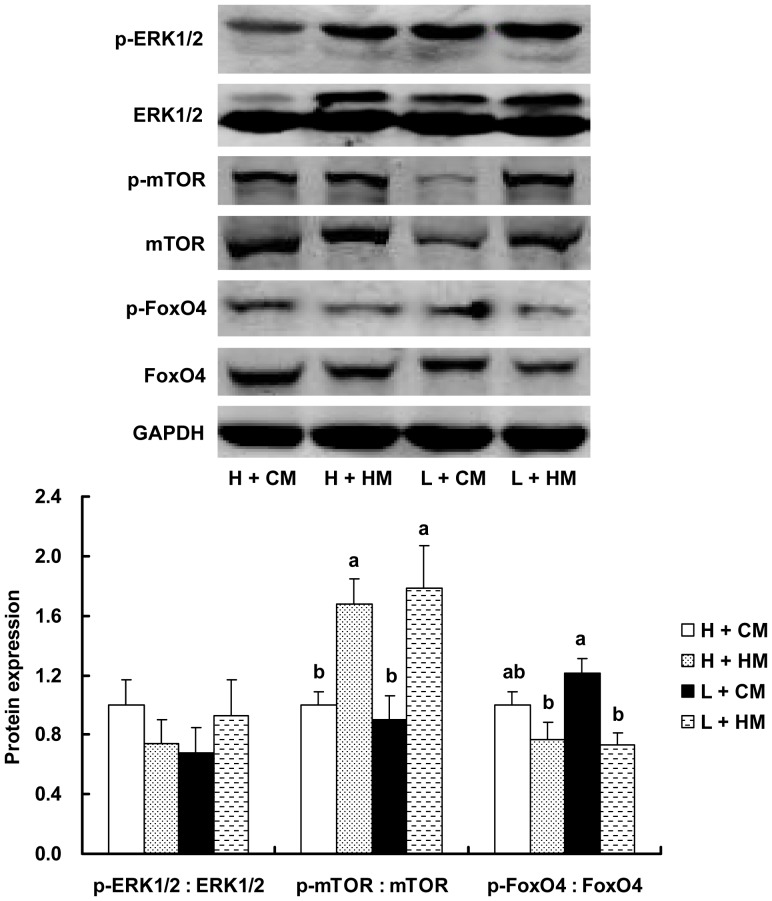
Phosphorylation of ERK1/2, mTOR and FoxO4. Western blot analysis (up) and quantification of the results (down) are shown. H - heavy (48.3±0.1 g); L - light (41.7±0.1 g); CM – control methionine levels (0.50% and 0.43% methionine during 1–21 d and 22–42 d, respectively); HM - high methionine levels (0.60% and 0.53% methionine during 1–21 d and 22–42 d, respectively). Different superscripts indicate significant differences (*P*<0.05).

There was no difference in phosphorylation of ERK1/2 among treatments (Figure 1). The phosphorylation of mTOR and FoxO4 did not differ when the broilers were fed the control diets. High Met diets promoted the phosphorylation of mTOR both in heavy and light chicks, and decreased the phosphorylation of FoxO4 only in light chicks.

## Discussion

The present study confirmed that light chicks had less BWG than heavy chicks when both were fed the control diets, which is consistent with the findings of previous studies [5], [24]. This may be partly due to less FI during starter period. High Met diets improved BWG and G:F of light chicks during finisher period without any change in FI, suggesting that increasing dietary Met levels promoted the growth of light chicks by improving feed efficiency but not by increasing feed consumption. No response observed in heavy chicks implied that the Met levels in the control diets were adequate for them.

In the present study, heavy chicks had greater breast muscle yield than light chicks at 42 d of age when both were fed the control diets, which is consistent with the data of Sklan et al. [5], although the dietary Met levels were not reported in their study. This may be attributed to larger, or even better developed organs and yolk sac of heavy chicks at hatch. In addition, heavy chicks had lower DNA content and higher RNA/DNA ratio in breast muscle, indicating that they had less cell population but higher tissue cell activity [25]. It has been reported that heavier chicks had greater muscle mass with more satellite cells that exhibited greater proliferative activity [5]. High Met diets increased breast muscle yield of light chicks, which was in agreement with the result of Hickling et al. [26]. This finding may be due to decreased muscle cell population but increased cell size and enhanced tissue cell activity as reflected by the changes in DNA content, protein/DNA and RNA/DNA ratios of breast muscle. Zhai et al. [12] reported that high Met diet (0.41 vs. 0.51% Met from 21 to 42 d of age) may increase breast muscle growth of broilers by activating several canonical pathways related to muscle development and enhancing sarcoplasmic hypertrophy. However, high Met diets did not affect breast muscle yield or its DNA, RNA and protein contents in heavy chicks, indicating that the response of breast muscle development to dietary Met was dependent on HW of chicks. Heavy chicks may have better developed breast muscles that are less responsive to the current high Met diets.

As a member of transforming growth factor-β family, MSTN has been identified as a critical autocrine/paracrine inhibitor of skeletal muscle growth [27]. In this study, decreased mRNA level associated with increased methylation of gene exon 1 region of MTSN were induced by high Met diets only in light chicks, which is consistent with the result of Liu et al. [9]. This data implied that Met may improve breast muscle growth of light chicks by promoting the DNA methylation of MSTN, thus inhibiting its mRNA expression. DNA methylation is a potent epigenetic repressor of transcription [28], and methyl groups transferred in DNA methylation reactions are ultimately derived from Met, therefore, high dietary Met intake might be expected to increase DNA methylation [29]. The lack of response to high Met diets in heavy chicks may be explained by its relatively higher DNA methylation and lower mRNA level of MSTN compared with those in light chicks, although only a trend was observed.

The ERK1/2 signaling pathway is indispensable for muscle cell proliferation, and its activation is required for myoblast terminal differentiation [30]. However, in this study the ERK1/2 phosphorylation did not differ among treatments, suggesting that high Met diets may not regulate breast muscle growth through the activation of ERK1/2. This may be due to the fact that ERK1/2 is required for myoblast proliferation but is dispensable for muscle gene expression and cell fusion [31]. Increased mTOR and decreased FoxO4 phosphorylation were observed in breast muscle of light chicks fed high Met diets. The mTOR and FoxO pathways have been demonstrated to play an important role in protein synthesis and degradation in skeletal muscle [32], therefore, the present data implies that Met may improve breast muscle growth of light chicks by enhancing protein synthesis and inhibiting protein degradation. Increased mTOR phosphorylation may be associated with downregulated MSTN mRNA expression, because mTOR signaling is a key target that accounts for myostatin function in skeletal muscle [7]. Decreased FoxO4 activation may also be attributed to lower MSTN expression as shown by Reed et al. [33], who reported that inhibition of FoxO transcriptional activity decreased MSTN expression in the muscles of mice. The reason for increased mTOR phosphorylation without any change in FoxO4 phosphorylation in heavy chicks needs further elucidation.

In conclusion, high Met diets improved growth performance and breast muscle yield of light but not heavy chicks, which was associated with decreased muscle cell population but enhanced tissue cell activity and increased cell size. Increased MSTN gene exon 1 methylation and mTOR phosphorylation as well as decreased MSTN mRNA level and FoxO4 phosphorylation were involved in this process.
